# Stochastic neighbor embedding as a tool for visualizing the encoding capability of magnetic resonance fingerprinting dictionaries

**DOI:** 10.1007/s10334-021-00963-8

**Published:** 2021-10-23

**Authors:** Kirsten Koolstra, Peter Börnert, Boudewijn P. F. Lelieveldt, Andrew Webb, Oleh Dzyubachyk

**Affiliations:** 1 Division of Image Processing, Department of Radiology, Leiden University Medical Center, Albinusdreef 2, 2333 ZA Leiden, The Netherlands; 2 C. J. Gorter Center for High Field MRI, Department of Radiology, Leiden University Medical Center, Albinusdreef 2, 2333 ZA Leiden, The Netherlands; 3 Philips Research Hamburg, Röntgenstrasse 24, 22335 Hamburg, Germany; 4 Intelligent Systems Department, Delft University of Technology, Mekelweg 4, 2628 CD Delft, The Netherlands; 5 Electron Microscopy Facility, Department of Cell and Chemical Biology, Leiden University Medical Center, Albinusdreef 2, 2333 ZA Leiden, The Netherlands

**Keywords:** Magnetic resonance fingerprinting, T-SNE, Dictionary visualization, Encoding capability

## Abstract

**Objective:**

To visualize the encoding capability of magnetic resonance fingerprinting (MRF) dictionaries.

**Materials and methods:**

High-dimensional MRF dictionaries were simulated and embedded into a lower-dimensional space using t-distributed stochastic neighbor embedding (t-SNE). The embeddings were visualized via colors as a surrogate for location in low-dimensional space. First, we illustrate this technique on three different MRF sequences. We then compare the resulting embeddings and the color-coded dictionary maps to these obtained with a singular value decomposition (SVD) dimensionality reduction technique. We validate the t-SNE approach with measures based on existing quantitative measures of encoding capability using the Euclidean distance. Finally, we use t-SNE to visualize MRF sequences resulting from an MRF sequence optimization algorithm.

**Results:**

t-SNE was able to show clear differences between the color-coded dictionary maps of three MRF sequences. SVD showed smaller differences between different sequences. These findings were confirmed by quantitative measures of encoding. t-SNE was also able to visualize differences in encoding capability between subsequent iterations of an MRF sequence optimization algorithm.

**Discussion:**

This visualization approach enables comparison of the encoding capability of different MRF sequences. This technique can be used as a confirmation tool in MRF sequence optimization.

**Supplementary Information:**

The online version contains supplementary material available at 10.1007/s10334-021-00963-8.

## Introduction

Magnetic resonance fingerprinting (MRF) is a rapid MRI technique that is used to estimate tissue relaxation times ($T_1$, $T_2$) and other MR-related parameters such as proton density ($M_0$) [1]. These parameters often reflect pathology such as inflammation (increased $T_1$) and neurodegeneration (reduced $T_2^*$). Unlike many other quantitative imaging techniques [2–4], MRF simultaneously encodes $T_1$ and $T_2$, such that the corresponding parameter maps can be obtained in an efficient manner. The simultaneous encoding is established through a variable flip angle pattern in the data acquisition process, which, if designed well, creates a characteristic signal evolution for each tissue in the human body. The $T_1$ and $T_2$ values for each voxel can be found by matching the measured signal curve to a pre-calculated dictionary containing the simulated signal evolutions as a function of the applied flip angle sequence for all possible ($T_1$, $T_2$) combinations.

The quality of the resulting parameter maps substantially depends on the underlying MRF flip angle sequence. Recent works have shown that flip angle pattern optimization can either improve the accuracy of parameter quantification or reduce the scan time that is needed to achieve the same accuracy [5, 6]. It is also known that increasing the length of the MRF sequence improves the accuracy of the parameter maps, in particular $T_2$ [6, 7]. Therefore, determining the optimal sequence or flip angle train is very important.

The process of optimizing a sequence is not straightforward due to the large solution space and the lack of well-established measures of encoding quality. Moreover, the optimal sequence may actually be different for each application, and therefore the application of interest and its constraints should ideally be taken into account. Sommer et al. [6] have shown how a Monte-Carlo type approach can be used to predict the encoding capability of different MRF sequences. The measures of encoding are based on the inner product between neighboring dictionary elements, and the distinction is made between local and global measures of encoding. Later, Cohen and Rosen [8] and Zhao et al. [5] formulated the sequence optimization problem as an inverse problem, allowing one to actually calculate the optimized sequence under certain constraints, using a dot product matrix as the encoding measure. These techniques show promising results in terms of optimized encoding power, but they often focus on a selected number of tissues and therefore provide little insight into how the encoding capability of the optimized sequence varies for different tissues in the entire dictionary.

In this work, we present a visual approach to judge the encoding capability of MRF sequences that provides insight into local as well as global capabilities of encoding. We analyze the encoding capability of an MRF sequence by looking at its corresponding MRF dictionary, describing the relevant signal evolutions for the application of interest, as was also done in Ref. [6]. We use the dimensionality reduction technique t-distributed stochastic neighbour embedding (t-SNE) [10] to transform the high-dimensional MRF dictionary into a 2D and a 3D space. The resulting low-dimensional representation of the MRF dictionary in this low-dimensional space is referred to as an *embedding*. The choice of t-SNE is motivated by its capacity to detect small differences in signals while preserving the manifold structure, which makes it particularly useful for analyzing data with nonlinear structure such as MRF dictionaries. The embedding of the MRF dictionary is then visualized as a color-coded dictionary map, based on which the local and global encoding capability (as described by Sommer et al. [6]) is examined. The color values in these maps are a surrogate for location in the low-dimensional space. This method provides a framework for comparing different MRF dictionaries and, hence, corresponding sequences. We first demonstrate how our visual representation of MRF dictionaries can be used to compare the encoding capability of three well-known MRF sequences. We then compare our results obtained with t-SNE to these obtained with a singular value decomposition (SVD). Even though SVD has not been used to visualize the encoding capability of MRF dictionaries before, it allows us to analyze the effect of a nonlinear dimensionality reduction technique compared to a linear one. We show that in 3D the differences in encoding capability observed with t-SNE are very similar to those observed with the SVD, while in 2D the differences are much more pronounced with t-SNE than with the SVD. Our visual observations are confirmed by standard quantitative measures of encoding power described in literature [8]. Finally, we show that t-SNE is sensitive enough to visualize differences in encoding capability between multiple iterations of an MRF sequence optimization algorithm.

## Materials and methods

### MRF dictionaries

**Fig. 1:**
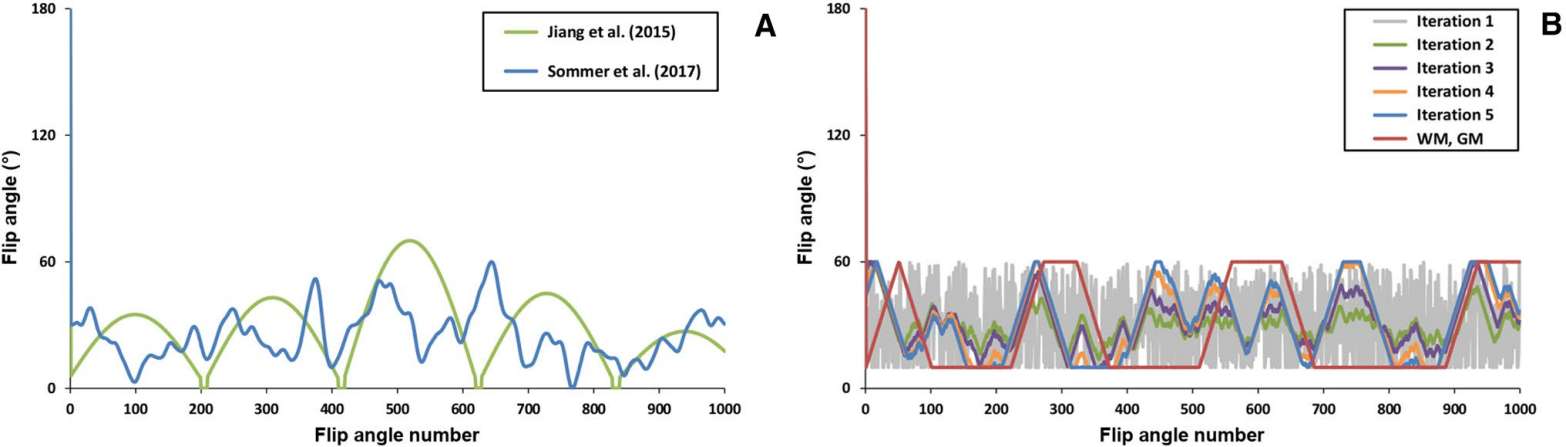
MRF flip angle patterns used. **A**  Smoothly varying pattern designed by Jiang et al. [11] (green line) and randomly varying pattern designed by Sommer et al. [6] (blue line). These sequences were used to create the corresponding dictionaries ${\mathcal {D}}_\text {J}$ and ${\mathcal {D}}_\text {S}$. Jiang’s pattern was also used to create a dictionary (${\mathcal {D}}_\text {J}^-$) without the preceding inversion pulse. **B**  Flip angle patterns for the first five iterations of a sequence optimization algorithm that optimizes the encoding between white matter and gray matter, and the final optimized sequence (red curve). These sequences were used to create the corresponding dictionaries ${\mathcal {D}}_{\text {O},i}^{\text {WM,GM}}$ for iteration *i*. All patterns start with an inversion pulse seen at flip angle number 0

Four different MRF flip angle sequences were used to generate five MRF dictionaries. All sequences consist of 1000 flip angles and a constant TR of 15 ms. The sequence shown in Fig. 1A contains a smoothly varying flip angle pattern introduced by Jiang et al. [11] (dictionary ${\mathcal {D}}_\text {J}$) and is preceded by a 180$^{\circ }$ inversion pulse. The same sequence was also analyzed without the inversion pulse (dictionary ${\mathcal {D}}_\text {J}^{-}$) to reduce the $T_1$ encoding ability. The third sequence constructed by Sommer et al. [6] (dictionary ${\mathcal {D}}_\text {S}$) has a more jagged random pattern; see also Fig. 1A. The fourth sequence was optimized for encoding between white matter (WM) and gray matter (GM), using a similar optimization technique to the one described by Cohen and Rosen [8]. The first five iterations and the final sequence (dictionary ${\mathcal {D}}_{\text {O},i}^{\text {WM,GM}}$, iteration *i*) are shown in Fig. 1B. The four MRF dictionaries were created by Bloch simulations using the extended phase graph formalism to model a fast imaging with steady state precession (FISP) sequence (unbalanced) [12]. $T_1$ values ranged from 20 to 2000 ms in steps of 30 ms and $T_2$ values ranged from 10 to 300 ms in steps of 10 ms. $B_1^+$ inhomogeneities were neglected. All dictionary calculations only included ($T_1$, $T_2$) combinations for which $T_1$ is larger than $T_2$.

### Dimensionality reduction

Each dictionary entry was reduced from 1000 to either 2 or 3 elements with t-SNE, which projects higher-dimensional data onto a lower-dimensional manifold while preserving similarity (pairwise distances) between data points. This approach is particularly useful for analyzing data with nonlinear structure such as MRF dictionaries. We used Barnes–Hut t-SNE [13] as the particular efficient implementation of t-SNE, which makes embedding of large data sets feasible in terms of computation time and allows us to analyze the dependence of the dimensionality on the obtained encoding capability. Embeddings were initialized with coordinates resulting from the principal component analysis. To ensure the convergence, the number of iterations was set to 1000. No additional data standardization (e.g. Z-scoring) was performed.

One of the parameters to tune in t-SNE is the perplexity parameter [9, 13] that influences formation of clusters in the embedding and is dependent on the size of the data set (number of dictionary entries). Therefore, its value was empirically set to the same value of 750 for 2D embeddings and 200 for 3D embeddings, based on an experiment in which we tested a range of realistic perplexity values. The final value was chosen as a “stable” one, in which range the embeddings effectively did not change.

Finally, ${\mathcal {D}}_\text {J}$, ${\mathcal {D}}_\text {S}$ and ${\mathcal {D}}_\text {J}^{-}$ were embedded in 2D (t-SNE-2D) up to 1000D (t-SNE-1000D) to investigate the dependence of the t-SNE dimensionality on the analyzed encoding capability.

### Embedding stability

To confirm reproducibility of the produced embeddings, we performed a stability experiment, similar to the one described by Dzyubachyk et al. [14]. Each of the dictionaries was embedded several times in 2D, using the aforementioned approach, and the results were compared to each other by registering them to the common reference embedding. In all cases, the embedding produced during the first execution (denoted as $\text {E}1$) was used as the reference. The distribution of point-wise distances between the corresponding dictionary entries in the low-dimensional embedding space was used as the quality measure. The minimal distance from each point in E1 to all the other points in this embedding is also calculated and used for comparison.

### Registration of embeddings

To facilitate comparison between different embeddings, they were mapped to a common reference frame, ensuring consistency of the color mapping. Without loss of generality, we selected the embedding $\text {E1}^{\text {(J)}}$ corresponding to the dictionary ${\mathcal {D}}_\text {J}$ as the reference for the MRF sequences. The registration was performed using a modification [15] of the Iterative Closest Point (ICP) algorithm [16] that also enables scale estimation. In this, we assumed that the correspondence between the point pairs is known, i.e. which embedding point was associated with which pair of ($T_1,T_2$) values, which allowed skipping the point matching step and significantly simplified the algorithm.

In the previous section, we demonstrated stability of ${\mathcal {D}}_\text {J}$, ${\mathcal {D}}_\text {S}$ and ${\mathcal {D}}_\text {J}^{-}$ by repeating the experiment that was described by Dzyubachyk et al. [14] for ${\mathcal {D}}_\text {J}$. This means that intrinsic stochastic effects resulting from using t-SNE can be neglected. In the same work, we also analyzed two ways of comparing two dictionaries: embedding them separately and jointly, in both cases followed by registration. Numerical results confirmed very similar performance of the two approaches, from which the conclusion was drawn that the former (separate embedding) is preferred for being faster. In this work, we used separate embedding, followed by registration, for all the MRF sequences.

### Color-coding of embeddings

For each dictionary, we mapped the coordinates of the low-dimensional embedding into the CIE L*a*b* color space [17]. Consequently, the color of each entry was mapped back to the dictionary space. In this way, a correspondence between each dictionary entry and a color was established, resulting in color-coded dictionary maps for each ($T_1$, $T_2$) combination. In these maps, similar colors indicate similar structure of the corresponding low-dimensional dictionary elements. From these color-coded dictionary maps the encoding capability as a function of $T_1$ and $T_2$ was visually analyzed.

### Quantitative measures

The low-dimensional embeddings, and therefore the color-coded dictionary maps, were validated using measures based on standard quantitative measures described in literature [8]. This was done in three steps. First, matrix *S*, describing the similarity of each dictionary entry with respect to all the other entries, was calculated for all the dictionaries. Since the Euclidean distance was used to optimize the t-SNE embeddings, this similarity matrix was defined as

1 $$\begin{aligned} S_{ij}=\Vert \mathbf{d }_i-\mathbf{d }_j\Vert _2. \end{aligned}$$

Note that for SVD the embedding entries $\mathbf{d }_i$ were first normalized. The similarity matrices were normalized to a maximum value of unity on its diagonal by rescaling *S* according to $S_{ij}=1-{S_{ij}}/{\text {max}_{ij} (S_{ij})}$. Second, only the entries in the similarity matrix corresponding to WM ($T_2 = 80~\text {ms}$) and GM ($T_2=110~\text {ms}$) were selected [18], resulting in two similarity matrices of reduced size: $S^{\text {WM}}$ $\in {\mathbb {R}}^{64\times 64}$ and $S^{\text {GM}}$ $\in {\mathbb {R}}^{63\times 63}$. Note that these similarity matrices describe the encoding along one vertical line (of constant $T_2$ values) in the color-coded dictionary maps, similar to those presented in Ref. [19]. Third, the distance between the similarity matrix and the identity matrix was used as a quantitative measure for WM and for GM:

2 $$\begin{aligned} \varepsilon _{\text {WM}}= & {} \frac{1}{M}\Vert I -S^{\text {WM}} \Vert _1 \qquad \text {and} \end{aligned}$$

3 $$\begin{aligned} \varepsilon _{\text {GM}}= & {} \frac{1}{N}\Vert I - S^{\text {GM}} \Vert _1. \end{aligned}$$

with $M=64$ and $N=63$ being the number of elements in each dimension of the corresponding similarity matrices. We use the normalized $\ell _1$ norm to assign a single number to the encoding capability of an embedding for a specific $T_2$ value. Note that these measures are low in case of a strong diagonal structure of $S^i$, indicating a good encoding capability for tissue *i*.

## Results

Online Resource 1 shows the results of the embedding stability experiment for the first three MRF dictionaries. These results clearly confirm high reproducibility of the embeddings of these dictionaries. In addition, Online Resource 2 shows that the embedding structures are stable in the analyzed range of perplexity values.

**Fig. 2:**
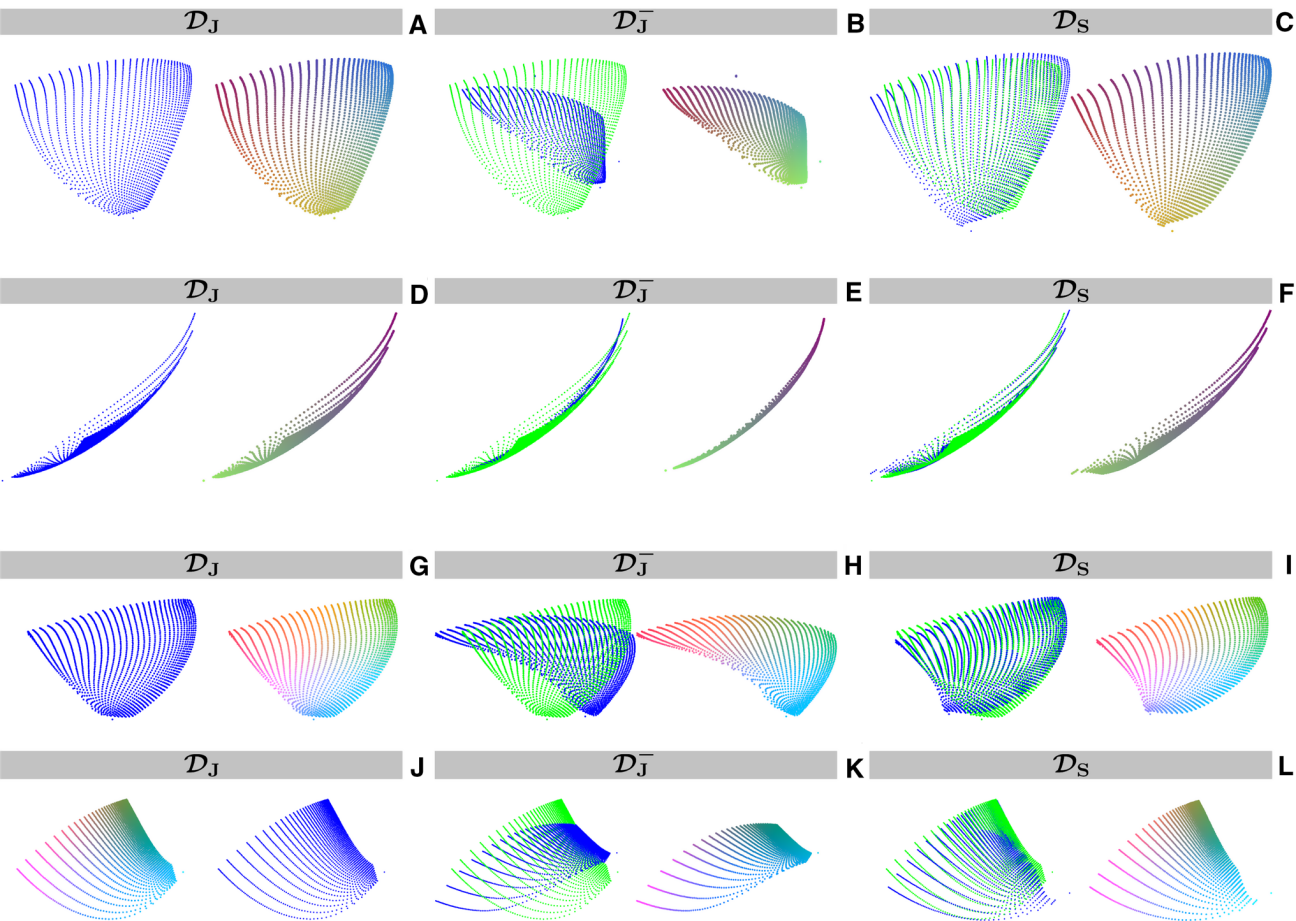
Comparison of 2D and 3D embeddings for three different MRF flip angle patterns. Two-dimensional embeddings of ${\mathcal {D}}_\text {J}$ (**A**,**D**), ${\mathcal {D}}_\text {J}^-$ (**B**,**E**) and ${\mathcal {D}}_\text {S}$ (**C**,**F**) produced by t-SNE (**A**–**C**) and SVD (**D**–**F**), respectively. The original embeddings (in blue) were registered to the corresponding embedding of ${\mathcal {D}}_\text {J}$ (in green). These embeddings are shown next to their colored counterparts. Embeddings for ${\mathcal {D}}_\text {J}$ and for ${\mathcal {D}}_\text {S}$ look very similar, while both being very different from that for ${\mathcal {D}}_\text {J}^-$. This can be observed both for t-SNE and for the SVD. Three-dimensional embeddings t-SNE-3D (**G**–**I**) and SVD-3D (**J**–**L**) are also shown

Figure 2 shows the 2D and 3D t-SNE embeddings and their color-coded counterparts for the dictionaries generated for the three MRF sequences: ${\mathcal {D}}_\text {J}$, ${\mathcal {D}}_\text {J}^-$ and ${\mathcal {D}}_\text {S}$. These sequences encode $T_1$ and $T_2$ simultaneously, resulting in embeddings that are two-dimensional and three-dimensional manifolds. The same figure shows, for comparison, the corresponding manifolds obtained with the SVD. The 2D embeddings corresponding to ${\mathcal {D}}_\text {J}$ and ${\mathcal {D}}_\text {S}$ are very similar, while being very different from that corresponding to ${\mathcal {D}}_\text {J}^-$. This is observed both for t-SNE and for the SVD. One can furthermore observe that the points in the t-SNE point clouds are more uniformly distributed than in the SVD point clouds, where most of the dictionary entries are clustered around the location represented by the green color. For the 3D t-SNE embeddings the difference with the SVD embeddings is smaller compared to that in 2D.

**Fig. 3:**
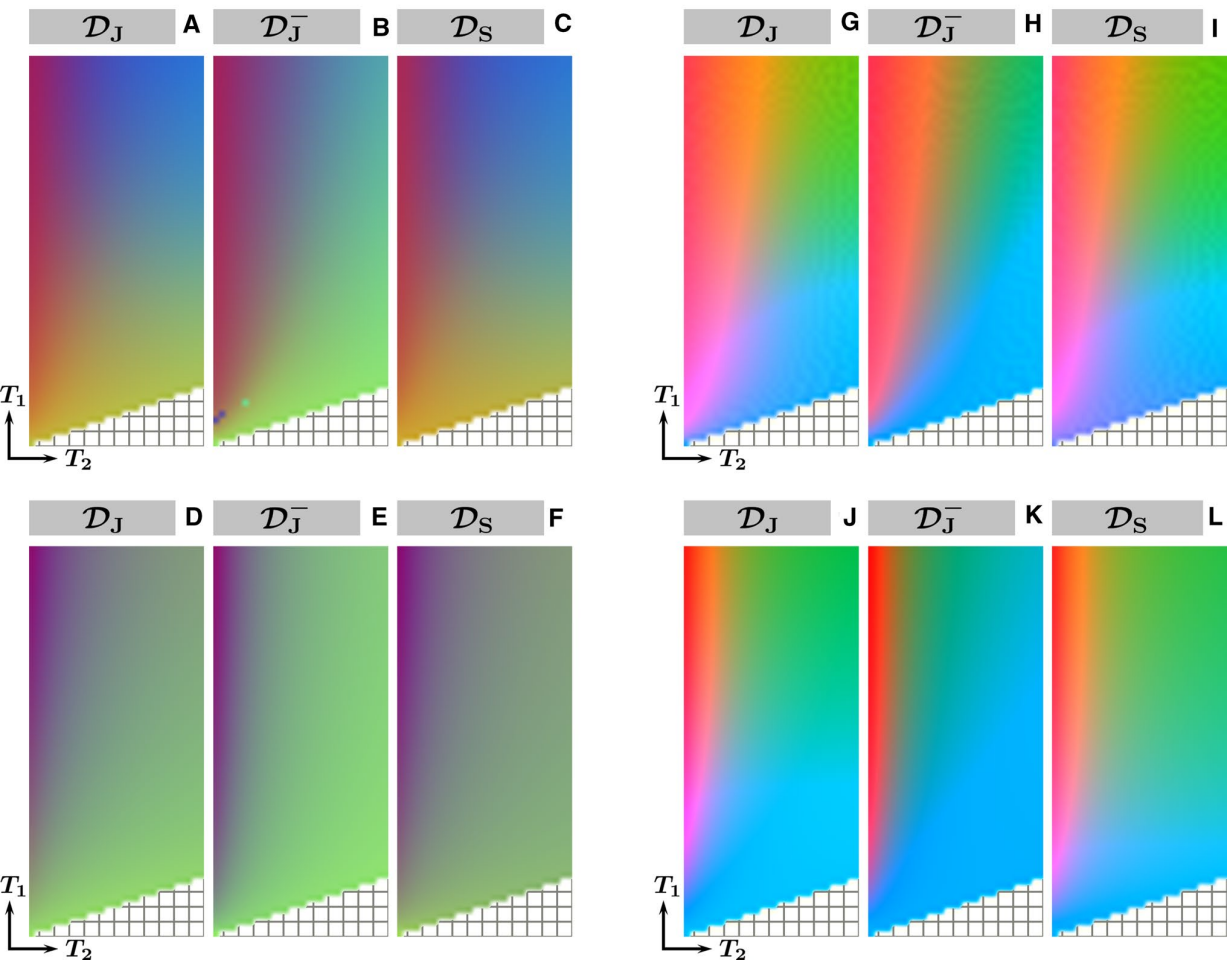
Comparison of the color-coded dictionary maps for three different MRF flip angle patterns embedded in 2D (left) and 3D (right). Two-dimensional color-coded dictionary maps corresponding to the embeddings of ${\mathcal {D}}_\text {J}$ (**A**,**D**), ${\mathcal {D}}_\text {J}^-$ (**B**,**E**) and ${\mathcal {D}}_\text {S}$ (**C**,**F**) in the ($T_1$, $T_2$) coordinate system. The embeddings were produced by either t-SNE (**A**–**C**) or SVD (**D**–**F**), respectively, and registered to that of ${\mathcal {D}}_\text {J}$ (see Fig. 2). In these maps, similar colors for certain ($T_1,T_2$) combinations indicate similar structure of the corresponding low-dimensional representations of the dictionary entries. Like the embeddings, also the color-coded dictionary maps for ${\mathcal {D}}_\text {J}$ and ${\mathcal {D}}_\text {S}$ look very similar, both for t-SNE and for the SVD, suggesting comparable encoding capability. The sequence without the inversion pulse results in a color-coded dictionary map with less color variation in the $T_1$ direction, especially for long $T_1$ values, suggesting reduced encoding capability compared to ${\mathcal {D}}_\text {J}$ and ${\mathcal {D}}_\text {S}$. This is much better visible with t-SNE than with the SVD. Similar results are also shown for t-SNE-3D (**G**–**I**) and SVD-3D (**J**–**L**). Note that figure (**B**) contains visible outliers caused by the stochastic nature of t-SNE, which can be mitigated by averaging the embeddings produced by repeated t-SNE runs. The pattern-filled triangle in the bottom of the color-coded dictionary maps represents the unsampled region for which $T_2$ is longer than $T_1$

Figure 3 shows the corresponding color-coded dictionary maps, from which the encoding capability as a function of $T_1$ and $T_2$ can be analyzed. Removing the inversion pulse from Jiang’s sequence reduces the encoding capability, which can be observed from the smaller color variation in the $T_1$ direction in its color-coded dictionary map, especially for long $T_1$ values. The color-coded dictionary maps for Sommer’s (${\mathcal {D}}_\text {S}$) and Jiang’s (${\mathcal {D}}_\text {J}$) sequences look very similar, suggesting that these sequences provide comparable encoding quality. Figure 3 also shows similar results for the color-coded dictionary maps produced with the SVD. In 2D, these maps show much less color variation compared to the color-coded dictionary maps produced with t-SNE. In 3D, however, the difference between the color-coded dictionary maps produced with SVD and with t-SNE is much smaller.

**Fig. 4:**
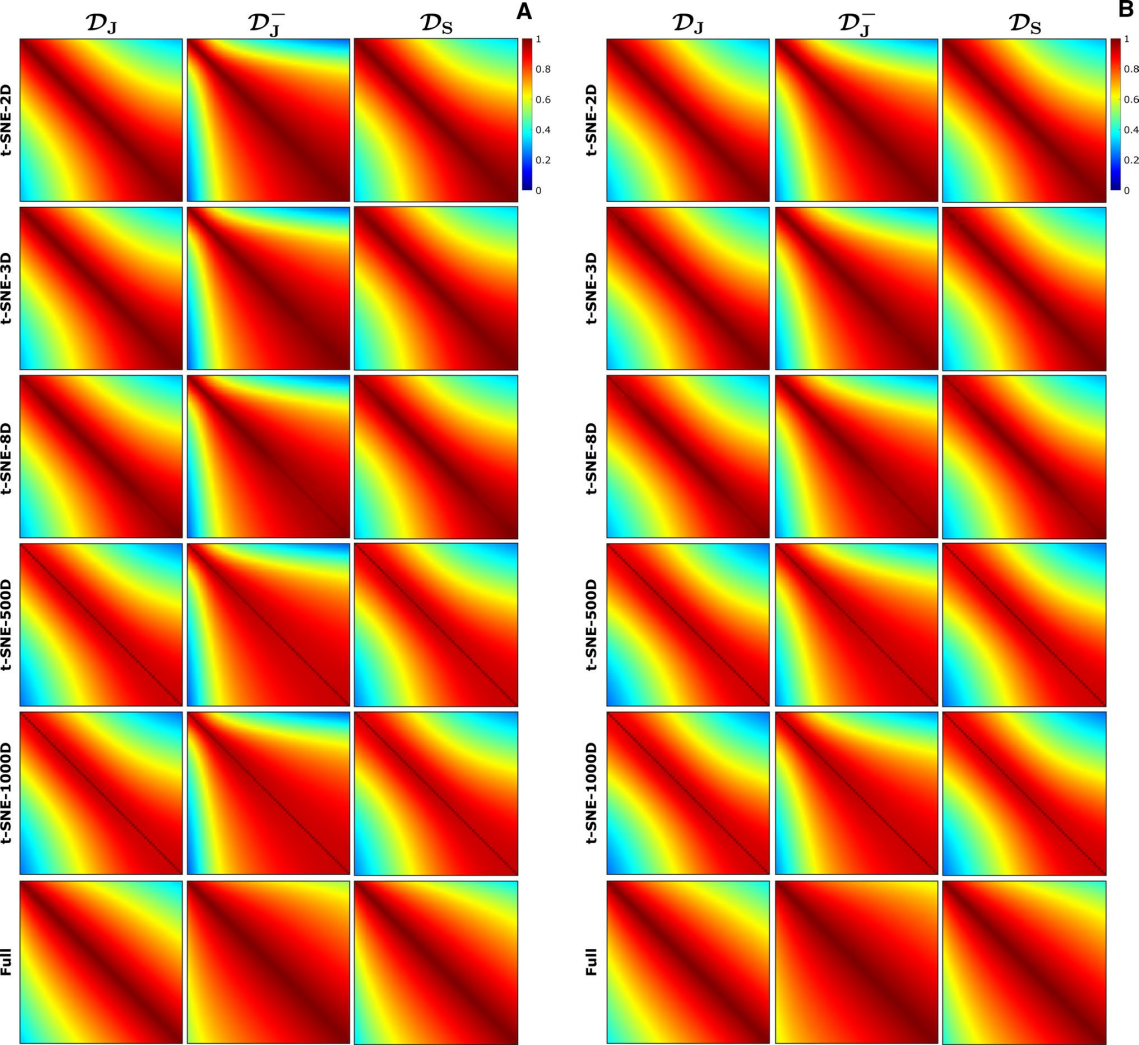
Similarity maps derived from the t-SNE embeddings for different dimensionalities. The similarity matrices were calculated for a fixed $T_2=80~\text {ms}$, corresponding to WM (**A**), and for a fixed $T_2=110~\text {ms}$, corresponding to GM (**B**), using the Euclidean distance as a similarity measure. The diagonal elements of the similarity matrices are equal to one by definition. The horizontal and vertical axes of each similarity map represent the different possible $T_1$ values corresponding to the fixed $T_2$ value. The similarity maps for WM and GM show very little dependency on the dimensionality (2–1000) of the t-SNE embedding. The nonlinear behavior of t-SNE can be observed from the difference between the similarity maps obtained from the full dictionaries and that obtained with t-SNE. For all dimensionalities, the similarity maps for ${\mathcal {D}}_\text {J}$ and ${\mathcal {D}}_\text {S}$ show a more diagonal structure than that of ${\mathcal {D}}_\text {J}^-$, both for WM and for GM, confirming a higher encoding capability for ${\mathcal {D}}_\text {J}$ and ${\mathcal {D}}_\text {S}$ than for ${\mathcal {D}}_\text {J}^-$

**Fig. 5:**
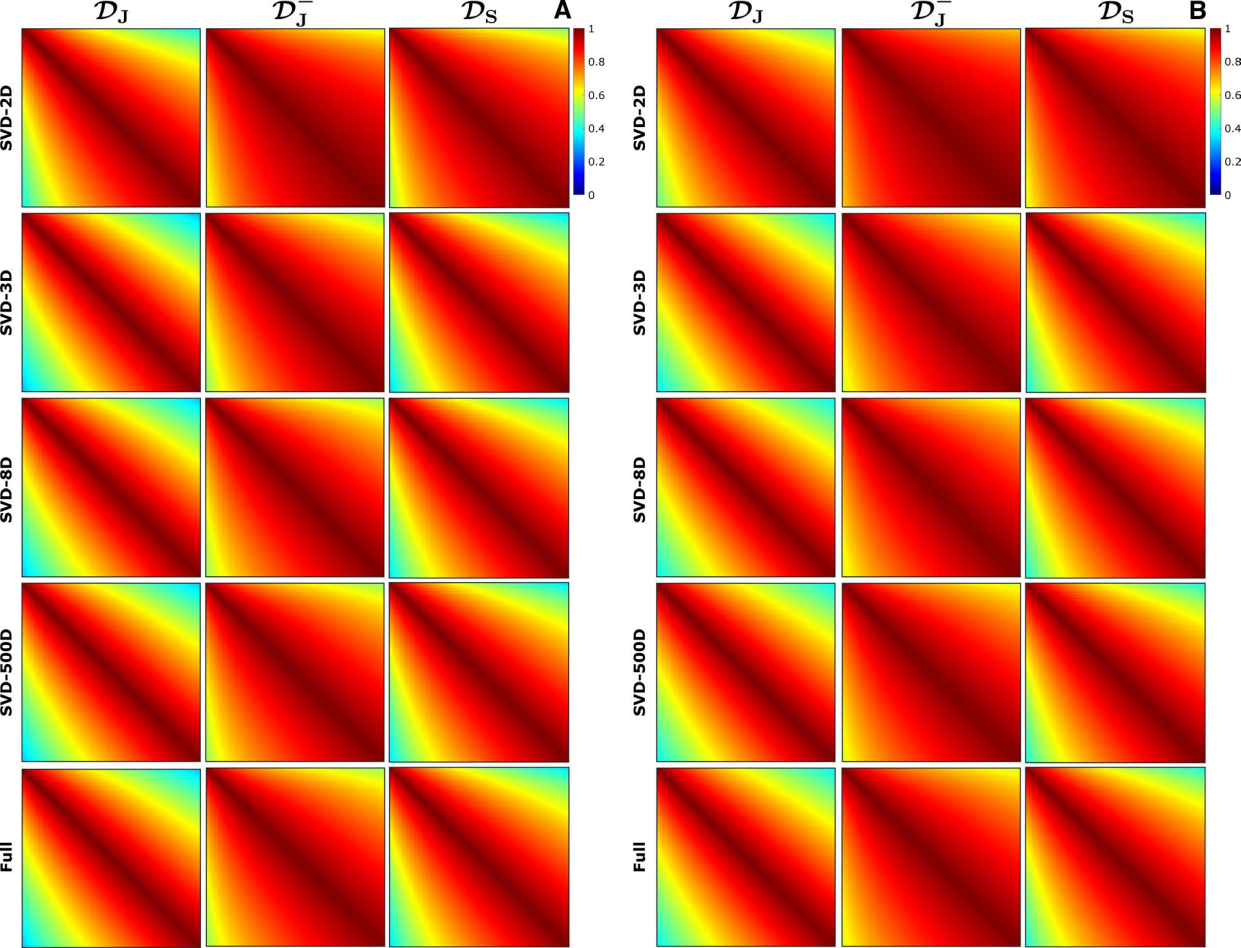
Similarity maps derived from the SVD embeddings for different dimensionalities. The similarity matrices were calculated for a fixed $T_2=80~\text {ms}$, corresponding to WM (**A**), and for a fixed $T_2=110~\text {ms}$, corresponding to GM (**B**), using the Euclidean distance as a similarity measure. The diagonal elements of the similarity matrices are equal to one by definition. The horizontal and vertical axes of each similarity map represent the different possible $T_1$ values corresponding to the fixed $T_2$ value. The similarity maps for WM obtained from the SVD embeddings are more dependent on the dimensionality (2D vs 3D) than these obtained from the t-SNE embeddings, shown in Fig. 4. The similarity maps obtained from the SVD embeddings show slightly less color variation compared to these obtained with t-SNE

Figure 4 shows the similarity maps derived from the low dimensional t-SNE embeddings using Eq. (1), for $T_2$ values corresponding to WM ($T_2$ = 80 ms) and to GM ($T_2$ = 110 ms). The similarity maps show minor differences when increasing the dimensionality of the embedding from 2D (t-SNE-2D) to 1000D (t-SNE-1000D), both for WM and for GM. The similarity maps for t-SNE-1000D show steeper patterns compared to that of the high-dimensional dictionaries analyzed without t-SNE, suggesting that the t-SNE analysis can increase the encoding capability of an MRF sequence. Similar results are shown in Fig. 5A for the low-dimensional SVD embeddings. In this case the difference between the similarity maps for dimensionalities 2D and 3D is larger compared to that for t-SNE, but the similarity map for the 500D embedding is closer to that of the high-dimensional dictionary compared to the one for the t-SNE case. Figure 5 shows, for all dimensionalities, a slightly smaller color variation within each SVD similarity map compared to these obtained with t-SNE. This indicates that the encoding capability of different MRF sequences is slightly better visualized with t-SNE than with SVD.

**Fig. 6:**
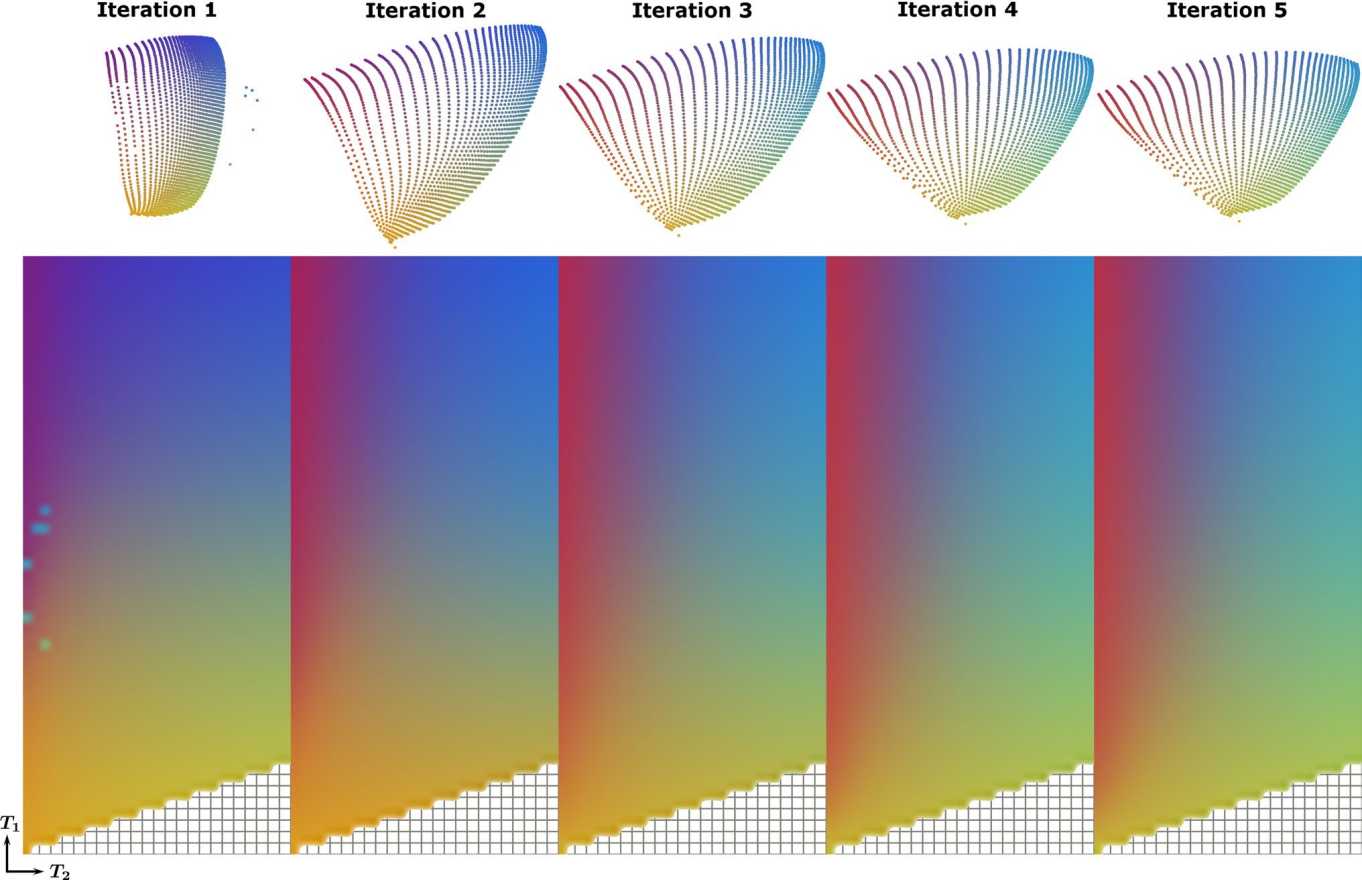
Comparison of the first five iterations of an MRF sequence optimization algorithm and their respective embeddings and color-coded dictionary maps. Two-dimensional t-SNE embeddings of ${\mathcal {D}}_{\text {O},i}^{\text {WM,GM}}$ were first registered to each other. The corresponding color-coded dictionary map shows a more gradual color change, especially in the $T_2$ direction, for the sequence in the fifth iteration compared to the sequence in the first iteration. Note that the color-coded map of the first iteration contains visible outliers caused by the stochastic nature of t-SNE, which can be mitigated by averaging the embeddings produced by repeated t-SNE runs. The pattern-filled triangle in the bottom of the color-coded dictionary maps represents the unsampled region for which $T_2$ is longer than $T_1$

**Fig. 7:**
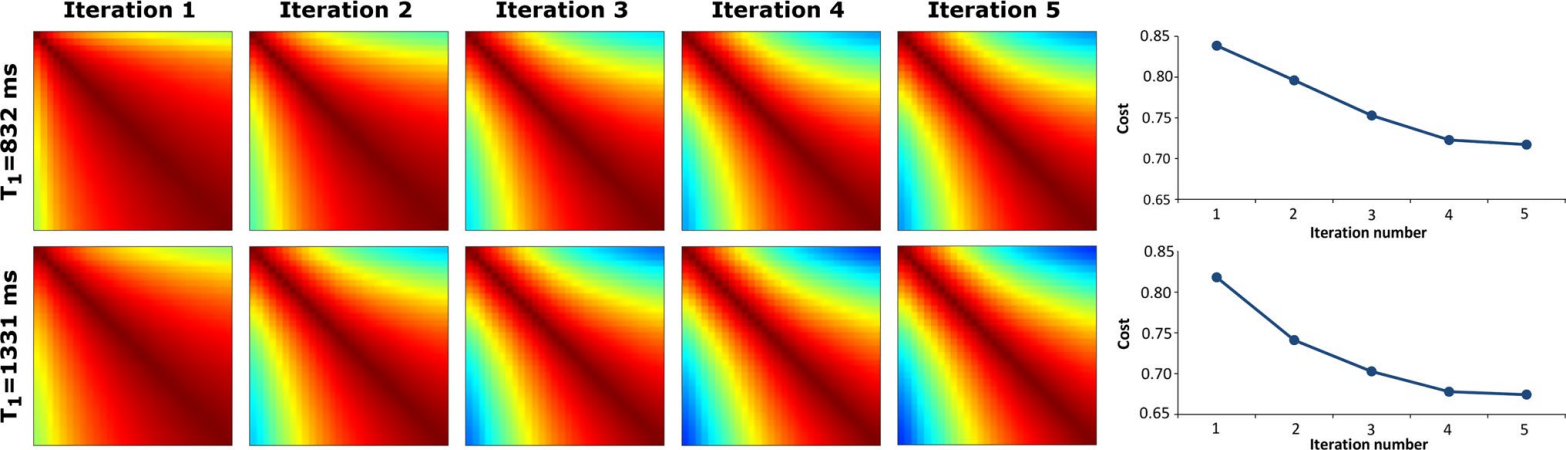
Comparison of the first five iterations of an MRF sequence optimization algorithm and their respective similarity maps and quantitative measures. Similarity maps were generated from the embeddings shown in Fig. 6 by fixing the $T_1$ value to 832 ms (WM) and 1331 ms (GM), respectively. The horizontal and vertical axis of the similarity maps represent different $T_2$ values. The maps show a steeper pattern as the iteration number increases, suggesting increased encoding capability. This is confirmed by the quantitative measures, calculated using Eq. 2, plotted as a function of the iteration number

**Table 1:** Quantitative measures of encoding capability for white matter (WM) and gray matter (GM) derived from t-SNE embeddings of different dimensionalities

Dictionary	t-SNE-2D	t-SNE-3D	t-SNE-8D	t-SNE-500D	t-SNE-1000D	Full
$T_2$ = 80 ms (WM)						
${\mathcal {D}}_\text {J}$	0.743	0.742	0.740	0.701	0.703	0.755
${\mathcal {D}}_\text {J}^{-}$	0.764	0.764	0.760	0.745	0.745	0.834
${\mathcal {D}}_\text {S}$	0.752	0.750	0.746	0.709	0.711	0.781
$T_2$ = 110 ms (GM)						
${\mathcal {D}}_\text {J}$	0.734	0.732	0.730	0.692	0.694	0.772
${\mathcal {D}}_\text {J}^{-}$	0.755	0.754	0.752	0.738	0.738	0.852
${\mathcal {D}}_\text {S}$	0.737	0.735	0.732	0.695	0.696	0.796

The measures were calculated from Eqs. 2 and  3. Larger numbers indicate lower encoding capability. The encoding capability measures are very similar for t-SNE-2D to t-SNE-1000D. The numbers in column “Full” were derived directly from the original high-dimensional dictionaries. These numbers suggest that the dictionaries analyzed with t-SNE have a higher encoding power than the full dictionary (without the t-SNE analysis). This is a result of the nonlinear behavior of t-SNE and is influenced by the perplexity parameter, as shown in Online Resource 3. Note, however, that the ranking of the encoding capability of the three sequences is preserved for both WM and GM

**Table 2:** Quantitative measures of encoding capability for white matter (WM) and gray matter (GM) derived from SVD embeddings of different dimensioalities

Dictionary	SVD-2D	SVD-3D	SVD-8D	SVD-500D	Full
$T_2$ = 80 ms (WM)					
${\mathcal {D}}_\text {J}$	0.796	0.752	0.755	0.755	0.755
${\mathcal {D}}_\text {J}^{-}$	0.868	0.835	0.833	0.834	0.834
${\mathcal {D}}_\text {S}$	0.843	0.778	0.781	0.781	0.781
$T_2$ = 110 ms (GM)					
${\mathcal {D}}_\text {J}$	0.821	0.769	0.772	0.772	0.772
${\mathcal {D}}_\text {J}^{-}$	0.890	0.853	0.852	0.852	0.852
${\mathcal {D}}_\text {S}$	0.868	0.793	0.795	0.796	0.796

The measures were calculated from Eqs. 2 and  3. Larger numbers indicate lower encoding capability. The encoding capability measures are very similar for SVD-3D to SVD-500D and for the full dictionaries. Note that the numbers in column “Full” were derived directly from the original high-dimensional dictionaries. The numbers are slightly different for SVD-2D than for higher dimensionalities, confirming that more than two dimensions are needed for SVD to capture the full encoding information

Tables 1 and 2 report quantitative measures of encoding capability derived from the similarity maps in Figs. 4 and  5 using Eq. (2). These numbers confirm that the encoding capability for ${\mathcal {D}}_\text {J}$ and ${\mathcal {D}}_\text {S}$ is comparable, whereas the encoding capability for ${\mathcal {D}}_\text {J}^-$ is lower, both for WM and for GM. This is the case for t-SNE and for SVD and for all dimensionalities. The quantitative measures for 2D SVD confirm that in this case the dimensionality reduction was too large to capture all the encoding information. The quantitative measures for the full dictionary (without the t-SNE analysis) are higher than these for t-SNE-1000D, even though the dimensionalities are the same for both cases. This difference is introduced by the nonlinear behavior of t-SNE, which is influenced by the choice of the perplexity value. Online Resource 3 shows that using lower perplexity values can reduce these differences, while having minimal effect on the color-coded dictionary maps. Note, furthermore, that the ranking of the three sequences is preserved both for WM and for GM. Figure 6 shows the color-coded dictionary maps for the first five iterations of a sequence optimization algorithm that optimizes the encoding between WM and GM. The color-coded dictionary map after the fifth iteration shows a more gradual color change in all directions (but especially in the $T_2$ direction) compared to that after the first iteration. This is confirmed by a lower distance between the similarity matrix and the identity matrix (see Eq. (2)) for fixed $T_1$ values of 1331 ms (WM) and 832 ms (GM), and, therefore, suggesting a higher encoding capability as the iteration number increases: 0.818/0.839 (iteration 1) vs 0.674/0.717 (iteration 5) for WM/GM. Note that ${\mathcal {D}}_\text {J}$, with a distance between the similarity matrix and the identity matrix of 0.743/0.734, had the worst encoding capability after five iterations for a $T_1$ of 1331/832 ms. Figure 7 shows the similarity maps for ${\mathcal {D}}_{\text {O},i}^{\text {WM,GM}}$ for the first five iterations of the optimization algorithm. The increasing steepness of the similarity pattern and the decreasing quantitative measures suggest that t-SNE may be able to capture the encoding capability in the context of MRF sequence optimization.

## Discussion

This work has shown the feasibility of using dimensionality reduction techniques such as t-SNE and SVD to visualize and compare the encoding capability of different MRF sequences. t-SNE and SVD both resulted in comparable color-coded dictionary maps for ${\mathcal {D}}_\text {J}$ and ${\mathcal {D}}_\text {S}$, while that for ${\mathcal {D}}_\text {J}^-$ suggested a lower encoding power. These visual results were confirmed by quantitative measures of encoding capability derived from the low-dimensional embeddings. The nonlinear behaviour of t-SNE emphasized small differences in encoding capability, leading in 2D space to a larger visual difference in color-coded dictionary maps for different sequences compared to when using the SVD. In 3D these visual differences were much smaller, although still present. t-SNE was therefore more effective in visualizing differences in the encoding capability of MRF sequences than the SVD.

As we have shown in Figs. 6 and 7, t-SNE is able to visualize differences in encoding capability for subsequent iterations of an MRF sequence optimization algorithm. This technique could therefore be useful in the context of MRF sequence optimization by providing visual information about the encoding capability of the optimized sequence for all the possible tissue parameters. Often only a few tissue types are taken into account in the optimization process to maintain computational efficiency. Since t-SNE emphasizes small differences in encoding capability, it could also potentially be of help in MRF sequence optimization when used as a dictionary transform in the cost function. The steeper similarity maps when analyzed with t-SNE, shown in Fig. 4, are in line with this hypothesis. Currently, calculation of a t-SNE embedding of one entire MRF dictionary ($1000\times 1855$ elements) takes a time that is in the order of minutes, from which we predict that embedding a limited number of selected tissues (needed during optimization) will take a time in the order of seconds. The feasibility of integrating t-SNE in an MRF sequence optimization algorithm will be explored in future work. In particular, we expect that GPU-based implementation of t-SNE [20] might be able to provide the required performance as it has been shown to speed-up the computations up to several orders of magnitude. Alternatively, one could think about using t-SNE as a compression tool before matching the MRF data to the dictionary, as is sometimes done with the SVD [23]. This could potentially increase the accuracy of matched ($T_1,T_2$) pairs for which the differences between elements are relatively small, such as for very long $T_1$ values. Such an approach would require a noise-insensitive t-SNE implementation or the use of another noise-insensitive nonlinear dimensionality reduction technique such as self-organizing maps (SOM) [24].

High-dimensional dictionaries can be transformed into any *n*-dimensional space using t-SNE for *n* smaller than the dynamic length of the dictionary. Natural choices enabling straightforward visualization are $n=2$ or $n=3$. In this work, all MRF dictionaries were preferably transformed into a 2D space, since 2D embeddings tend to be more stable. Choosing $n>3$ would complicate visualization, but results in this work have already shown that the dependence of the dimensionality ($n=\overline{21,000}$) on the quantitative encoding measures (distance between the similarity matrix and the identity matrix) is relatively small for t-SNE. When three encoding parameters (among which $T_1$ and $T_2$) are analyzed, the low dimensional embedding should be mapped to a 3D color-coded dictionary map instead of a 2D map. Online Resource 4 shows how this works for MRF dictionaries with $T_1$, $T_2$ and $B_1^+$ encoding. The same procedure could be used to analyze for example $B_0$ encoding for balanced SSFP sequences. If adding an extra encoding parameter brings a highly repetitive shape to the dictionary landscape, low-dimensional dictionary elements will show up close together in the embedding space, as was observed for low $B_1^+$ fractions. When more than three encoding parameters are analyzed at the same time, the visualization should be split into multiple 3D visualizations by sequentially selecting 3D subvolumes from the color-coded dictionary map.

Although in this study t-SNE was used to transform the high-dimensional dictionaries into low-dimensional space, there are many other nonlinear dimensionality reduction techniques that could be used instead. For several benchmark data sets, t-SNE has been shown to produce results of superior quality compared to other non-linear transformations such as Isomap and Locally Linear Embedding [10]. However, these results need to be reevaluated for MRF dictionaries to find out whether these conclusions hold in the context of quantitative MR sequences. Furthermore, the small differences between 3D t-SNE and 3D SVD for the currently analyzed dictionaries suggest that linear dimensionality reduction techniques could also provide useful information about the encoding capability for visualization purposes.

In this work, we have used existing measures of encoding capability described in literature to validate the visual results obtained with t-SNE. The question of which quantitative measures are suitable for describing such differences most efficiently or which measures describe the encoding capability best, is still open.

There are many design choices when creating MRF dictionaries. Differences introduced by these design choices may influence the visualization of the encoding capability of the dictionaries. As shown in Online Resource 4, color-coded dictionary maps and corresponding similarity maps for coarse and fine dictionaries look rather similar. Any small differences are likely caused by the need for choosing a different perplexity parameter due to an increased number of elements for the fine dictionary. Introducing a larger $T_2$ range results in a mismatch between the structure of the low- and the high-dimensional dictionaries, represented by the differently changing patterns in the similarity maps. This effect can be avoided by introducing a nonuniform step size in the $T_2$ dimension, to account for the intrinsic nonlinear behavior of the MRF data (i.e. dictionary entries for long $T_2$ values are much more similar to each other than dictionary entries for short $T_2$ values). These results suggest that the dictionaries should be constructed on the same grid when comparing dictionaries with t-SNE. Using a non-uniform step size in the $T_1$ dimension may also eliminate the locally steeper pattern in the similarity maps of Jiang’s sequence without inversion pulse, that we observe for very short $T_1$ values, compared to the other two sequences. MRF sequences of different length (i.e. different number of flip angles) can easily be compared by zero-padding the shorter sequence as was shown by Dzyubachyk et al. [14].

In this work, we embedded each sequence individually, after which the point clouds were registered to each other using a modification of the Iterative Closest Point algorithm with integrated scale estimation [15]. An alternative approach would be to first combine two dictionaries and treat them as one large dictionary in the embedding process, after which registration of the two point clouds corresponding to each of the combined dictionaries can be performed. As demonstrated earlier [14], both approaches provide comparable registration results, and hence result in similar color-coded dictionary maps. Since the latter approach is computationally more expensive due to the two-fold increase in the number of high-dimensional data points, the former approach is more attractive in this application.

Since the t-SNE algorithm is intrinsically stochastic, the final embeddings may in general differ from run to run. Dzyubachyk et al. [14] performed a quantitative stability study on Jiang’s dictionary [11] and the final embedding was highly reproducible. Here we repeated that stability study for different dictionaries used in this paper and a different implementation of t-SNE and quantitatively concluded high similarity between the results corresponding to different runs of the algorithm.

In conclusion, t-SNE can be used to visualize the encoding capability of entire MRF dictionaries. This technique can be used to obtain insight into the encoding principles by comparing different sequences, or as a confirmation tool in the context of MRF sequence optimization. This may support the use of this technique in clinical applications.

## Supplementary Information

Below is the link to the electronic supplementary material.

Supplementary file1 (PDF 5415 KB)
