# Supplementary material for: Stochastic neighbor embedding as a tool for visualizing the encoding capability of magnetic resonance fingerprinting dictionaries
Source: MAGMA. 2021 Oct 23;35(2):223–34. doi: 10.1007/s10334-021-00963-8 (PMC8995272; doi:10.1007/s10334-021-00963-8)
Supplement: Supplementary file 1 — Supplementary file1 (PDF 5415 KB) [file 10334_2021_963_MOESM1_ESM.pdf]

Online Resources

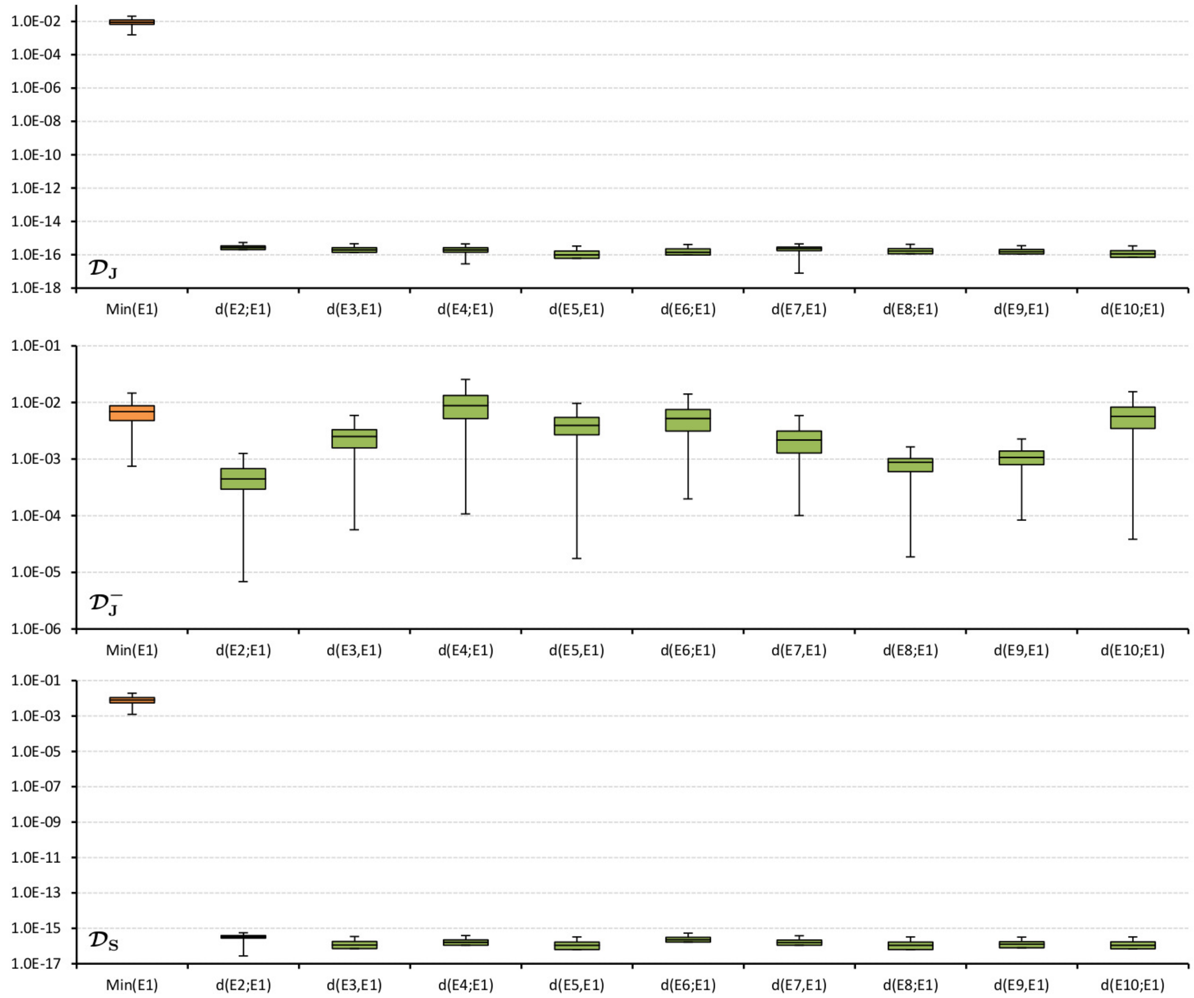

Online Resource 1: Stability of the 2D embeddings of three different MRF dictionaries. Label “Min(E1)” (orange box) denotes the distribution of the minimum distance between each point of E1 and all the other points in this embedding. Labels “d(E2;E1)” to “d(E10;E1)” (green boxes) denote distributions of the distances between the corresponding points in E1 and En (n=$\overline{2,10}$). Whiskers indicate the maximum and the minimum of the corresponding distribution. The plots indicate high repeatability of the embedding with negligible stochastic effects.

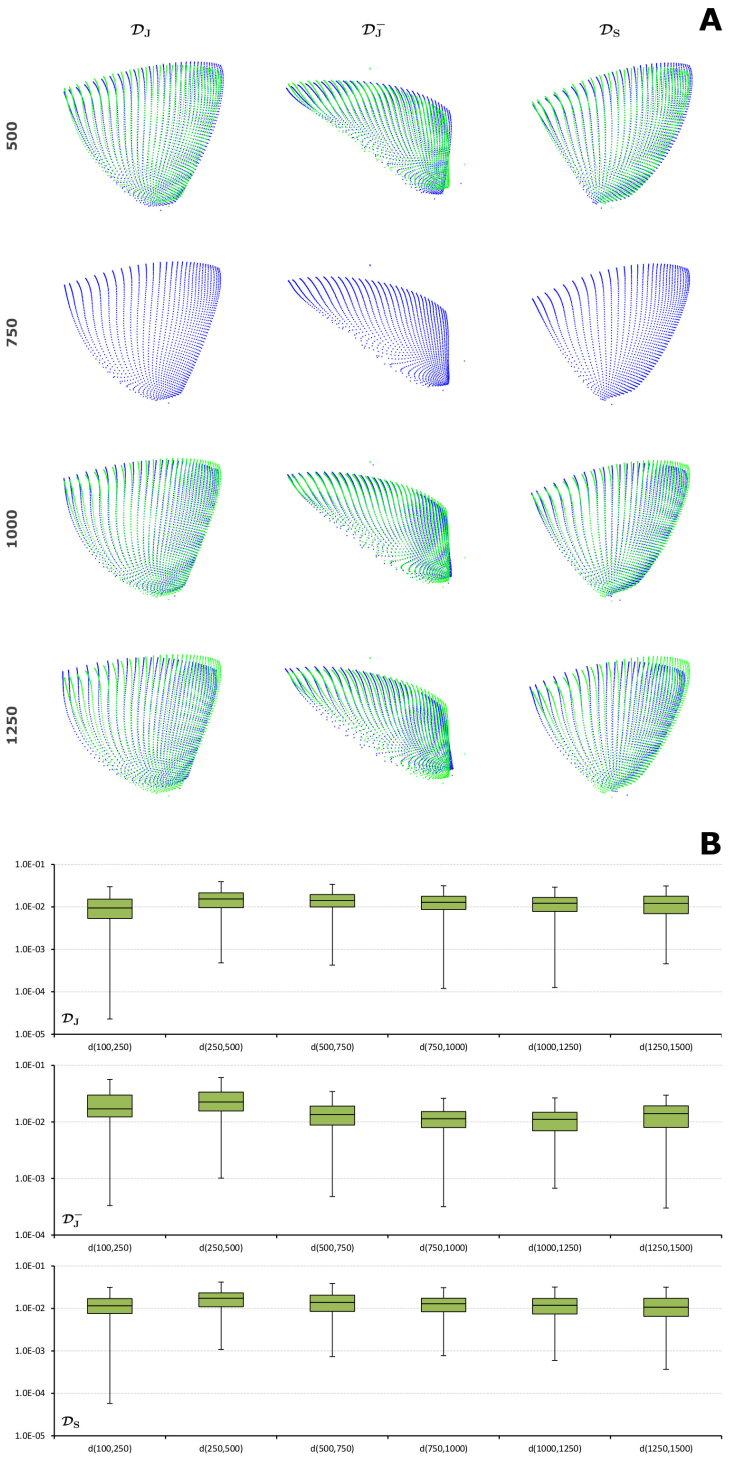

Online Resource 2: Scatter plots of the t-SNE embeddings of the three MRF dictionaries ($\mathcal{D}_\mathrm{J}$, $\mathcal{D}_\mathrm{J}^-$ and $\mathcal{D}_\mathrm{S}$) for different perplexity values. (**A**) Embeddings (depicted in blue) were registered to the corresponding embedding of the same dictionary calculated with a perplexity value of 750 (depicted in green). Embeddings corresponding to different perplexity values are very similar. (**B**) Boxplots representing distributions of the point-wise difference between each embedding and the one obtained with the neighboring perplexity value. These differences are in the same range for all perplexity values, meaning that the embeddings stabilize around the perplexity value of 750.

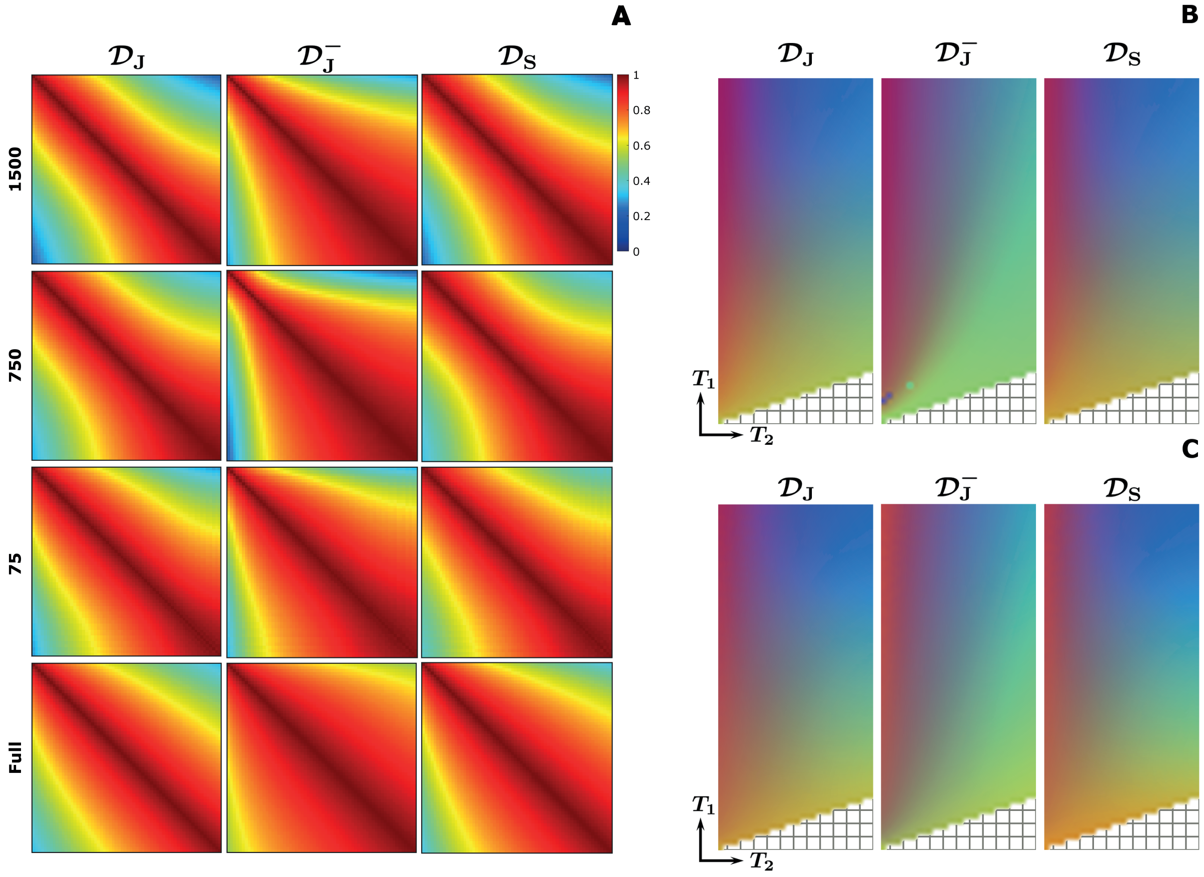

Online Resource 3: Comparison of similarity maps and color-coded dictionary maps for different perplexity values and a T_2 of 80 ms. (**A**) Lower perplexity values result in similarity maps with similar shape to these of the full dictionary, not analyzed with t-SNE. Perplexity values lower than 75 result in unstable t-SNE embeddings, showing tears in the t-SNE point clouds. To capture more global relations between the dictionary entries, slightly higher perplexity values are preferred. The color-coded dictionary maps for perplexity values of 750 (**B**) and 75 (**C**) show negligible differences.

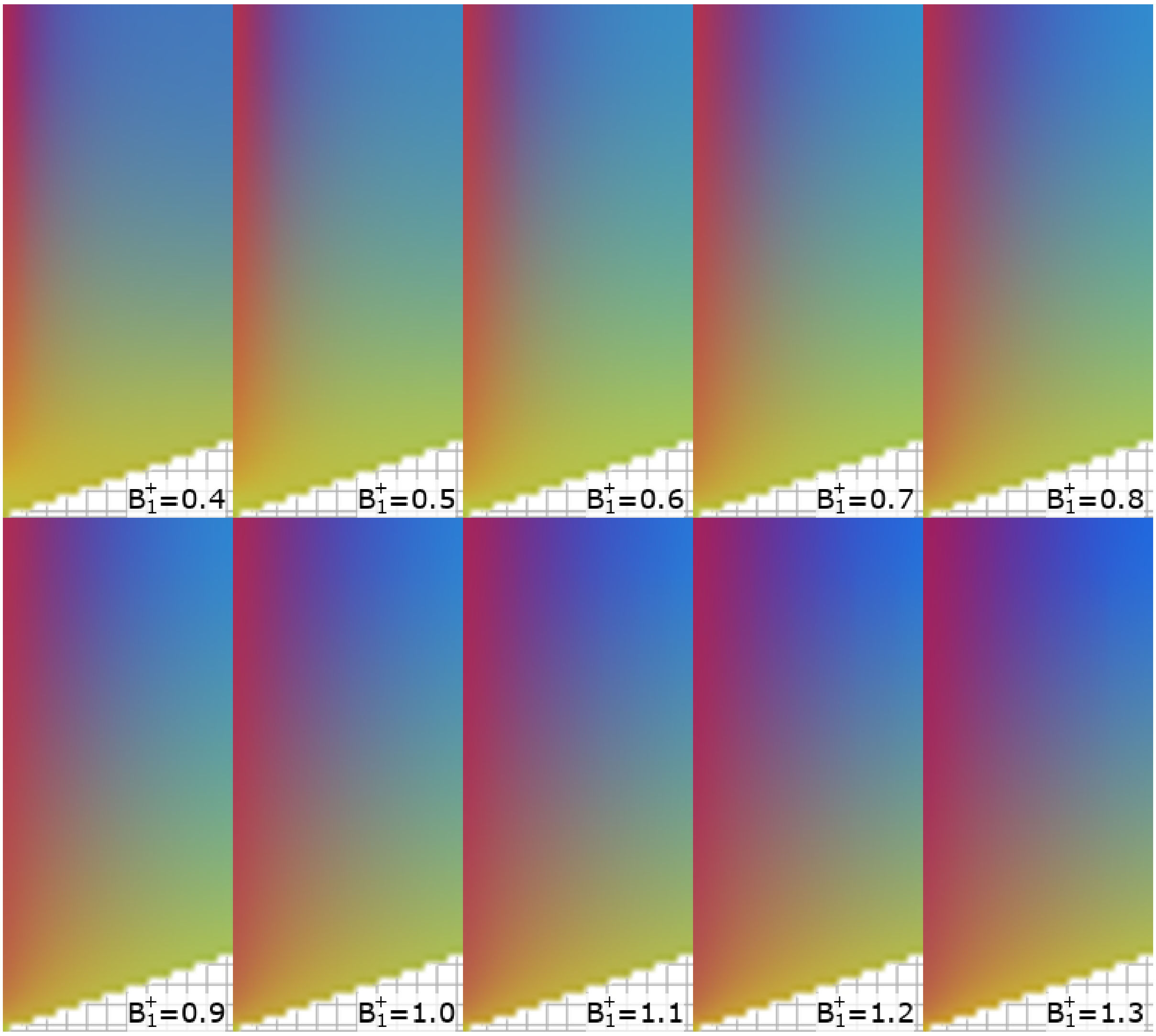

Online Resource 4: Comparison of different B_1^+ scaling factors embedded together. Two-dimensional embedding of $\mathcal{D}_\mathrm{J}$ including T_1, T_2 and B_1^+ scaling factors ranging from 0.4 to 1.3. The color-coded dictionary maps in the (T_1, T_2) coordinate system show that, in general, different B_1^+ fractions in the dictionary are represented by different shades of blue in the color-coded dictionary maps, suggesting that the B_1^+ map can be estimated in the matching process together with the T_1 and T_2 maps. Some regions, such as represented by short T_1 and/or T_2, show very similar colors for different B_1^+ fractions, suggesting that these (T_1, T_2, B_1^+) combinations are less well-encoded by this particular MRF sequence. The white triangle in the bottom of the color-coded dictionary maps represents the region for which T_2 is larger than T_1.

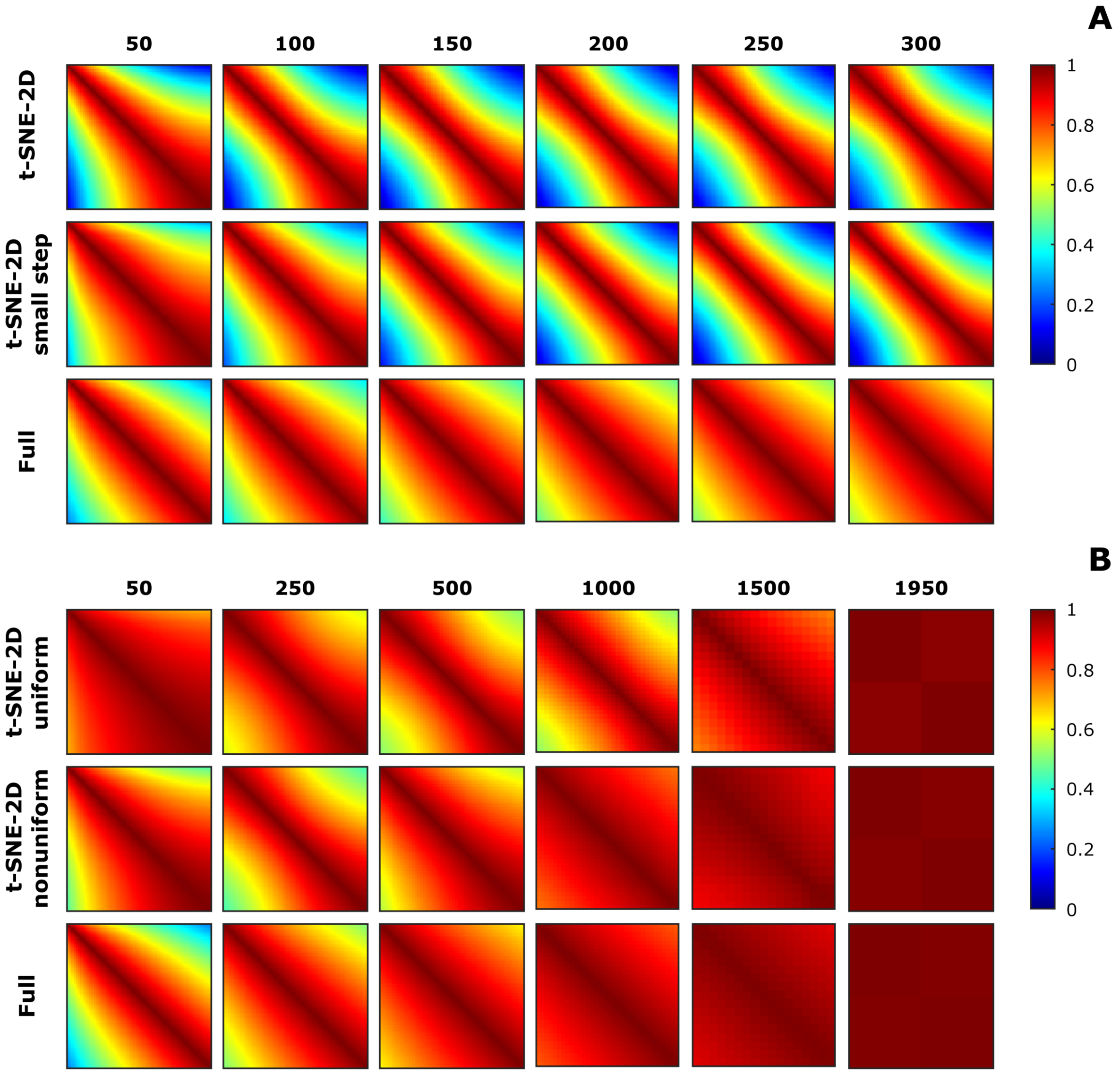

Online Resource 5: Comparison of similarity maps and color-coded dictionary maps for $\mathcal{D}_\text{J}$ with different T_2 range and step size. Similarity maps and color-coded dictionary maps for $T_2^{\text{max}} = 300$ ms (**A**) and for T_2^{max}=2000 ms (**B**), respectively. The horizontal and vertical axis of the similarity maps represent different possible T_1 values corresponding to the fixed T_2 value. (**A**) For the $T_2^{\text{max}} = 300$ ms, a uniform step size results in a good agreement between similarity map patterns obtained from the full dictionary and from the 2D t-SNE embedding. Using a twice as high T_1 and T_2 resolution ("t-SNE-2D small step") results in very similar similarity maps compared to the original dictionary. (**B**) Using a larger $T_2^{\text{max}} = 2000$ ms results in a mismatch between high ("Full") and low-dimensional ("t-SNE-2D uniform") similarity maps. Using a non-uniform step size in the T_2 dimension can help to resolve this mismatch ("t-SNE-2D nonuniform"). This suggests that the dictionaries should be constructed on the same T_1/T_2 grid when comparing dictionaries with t-SNE.
